# Memory dependent modulation of hippocampal theta power through frontoparietal phase synchronous brain stimulation

**DOI:** 10.1038/s41598-025-09841-y

**Published:** 2025-07-21

**Authors:** Nina M. Ehrhardt, Jevri Hanna, Dayana Hayek, Robert Fleischmann, Ulrike Grittner, Axel Thielscher, Agnes Flöel, Daria Antonenko

**Affiliations:** 1 Department of Neurology, University Medicine Greifswald, Ferdinand-Sauerbruch-Straße, 17475 Greifswald, Germany; 2 Berlin Institute of Health (BIH), Anna-Louisa-Karsch-Straße 2, 10178 Berlin, Germany; 3 Institute of Biometry and Clinical Epidemiology, Charité – University Medicine Berlin, Charitéplatz 1, 10117 Berlin, Germany; 4 Section for Magnetic Resonance, Department of Health Technology, Technical University of Denmark, Kgs Lyngby, Denmark; 5 Danish Research Centre for Magnetic Resonance, Centre for Functional and Diagnostic Imaging and Research, Copenhagen University Hospital Amager and Hvidovre, Copenhagen, Denmark; 6 German Centre for Neurodegenerative Diseases (DZNE) Standort Greifswald, Greifswald, Germany

**Keywords:** Neuroscience, Learning and memory

## Abstract

Targeting subcortical regions like the hippocampus with non-invasive brain stimulation is challenging due to predominantly cortical distribution of electric fields. Frontoparietal phase-synchronous transcranial alternating stimulation (tACS) offers a promising approach to modulate hippocampal functions, though its ability to induce modulation of oscillatory activity in deep subcortical targets remains to be shown. In this pilot study, we applied dual-site theta-tACS (in-phase between left frontoparietal regions) during a temporal order memory task. To quantify hippocampal activity and connectivity, we performed subcortical source analyses of electroencephalography (EEG) data using a hierarchical subspace pursuit algorithm. While memory did not differ between tACS and sham conditions, we observed tACS-induced increases in hippocampal theta power and its connectivity in individuals with superior memory performance. This study provides first-time evidence that phase-synchronized tACS over lateral frontoparietal areas influences hippocampal theta oscillatory activity, substantiating its potential to modulate hippocampal functions in health and disease.

**Supplementary Information:**

The online version contains supplementary material available at 10.1038/s41598-025-09841-y.

## Introduction

Cognitive processes rely on synchronized activity of large-scale, distributed brain networks. Theta-range neural oscillations are considered fundamental to episodic memory function in the hippocampal-cortical network^1,2^.

Targeting subcortical regions like the hippocampus with non-invasive brain stimulation presents challenges^3,4^ as the electric fields generated by tACS are predominantly distributed across the cortical surface^5–7^. Targeting cortical sites within the episodic memory network may offer a gateway into modulating memory functions that rely on deeper brain structures^8–11^. This has been shown in studies using transcranial magnetic stimulation (TMS)^12,13^ and transcranial direct current stimulation (tDCS)^14–17^. In this context, applying currents primarily over parietal brain regions has been shown to enhance functional connectivity between the stimulation targets and the hippocampus, supporting the observed improvements in memory^12,18^.

Previous studies have reported beneficial effects of phase-synchronous dual-site tACS on executive and working memory functions^19–24^. Inducing frontoparietal phase-synchrony in the theta band (4 to 8 Hz) through transcranial alternating stimulation (tACS) may also offer a promising approach for modulating episodic memory functions which are mediated by the hippocampal-cortical network^25^ though its ability to induce oscillatory modulation in deep subcortical targets remains to be shown.

To investigate whether hippocampal oscillatory electroencephalography (EEG) activity is modulated via dual-site tACS, we conducted a double-blind, counterbalanced crossover pilot study. In-phase (0° phase-lag) and sham theta-tACS (6 Hz) were applied to the left frontal and parietal regions of 20 healthy young participants (aged 19–29 years) during a sequence memory task performance, previously shown to be mediated by the left hippocampus^9^. A 64-channel electroencephalogram (EEG) was recorded to perform source analyses. To quantify theta power in the hippocampus and cortico-hippocampal connectivity, we used a hierarchical subspace pursuit algorithm^26^ that allows the estimation of subcortical sources by restricting cortical activity to a sparse set of cortical sources^27^.

## Results and discussion

In this pilot study, we administered focal dual-site in-phase tACS over left inferior frontal and posterior parietal regions. Our aim was to investigate hippocampal oscillatory EEG activity as a function of stimulation condition, by quantifying hippocampal theta power and cortico-hippocampal connectivity.

### Memory performance

**Fig. 1:**
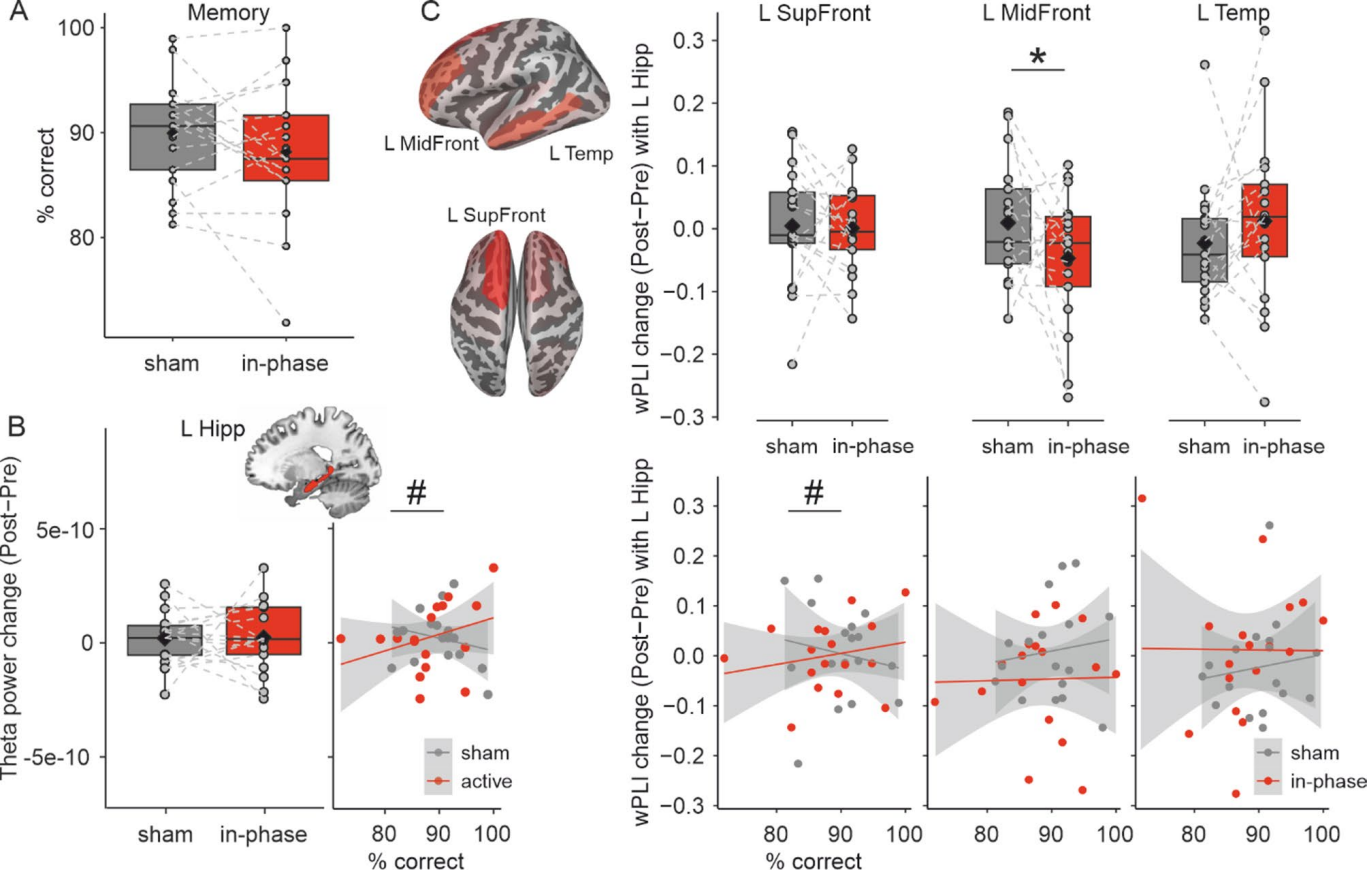
Hippocampal network involvement in fronto-posterior tACS. Our findings showed (**A**) no evidence for a beneficial effect of in-phase tACS on memory performance, (**B**) no evidence for a main effect of stimulation on left hippocampal theta power (left), but increased theta power for participants with superior memory performance (right), and (**C**) an interaction with memory performance on wPLI between left hippocampus and left superior frontal cortex, such that wPLI increased for those with superior memory performance (bottom left), and a main effect of stimulation on wPLI between left hippocampus and left middle frontal cortex (top middle). Boxplots show individual data points with the mean (diamond), median (vertical line), 25th and 75th percentiles (lower and upper hinges), and 1.5*interquartile range (lower and upper whiskers). Scatterplots show individual data points and a linear regression line with 95% confidence interval. All analyses included data of 17 participants. L MidFront, left middle frontal. L SupFront, left superior frontal. L Temp, left temporal. L Hipp, left hippocampus. wPLI, weighted phase-lag index. 80%-CI does not include 0: * main effect # interaction effect.

With regard to memory performance in the temporal order memory task, no overall memory performance differences were found between the theta-tACS and sham condition (mean difference between conditions: −1.66, 80% CI = −3.45 to 0.12, semi-partial *R*² = 0.03, Fig. 1A), indicating heterogeneous effects across individuals. The absence of a group-level behavioral difference may reflect interindividual variability, ceiling effects in memory of our group of healthy young adults, or the potential requirement of a higher dose (e.g., more sessions or greater intensity) to elicit measurable behavioral changes. Previous research on memory effects in healthy adults has yielded mixed results, with some studies showing improvement while others did not^28^. Studies that have applied frontoparietal phase-synchronous tACS to modulate working memory functions, have reported benefits for theta-tACS compared to sham^22,24^. Whether it can improve episodic memory functions remains to be investigated in future studies. Importantly, the absence of a group-level behavioral difference does not necessarily imply the absence of an effect of stimulation, as considerable variability among individual responsiveness has been shown on both behavioral and neuronal levels with several internal and external factors influencing whether an individual will respond on the behavioral level to brain stimulation or not^24,29–31^.

## Hippocampal theta power

With regard to brain activity, previous studies using functional magnetic resonance imaging (fMRI) have shown activity modulations through brain stimulation in regions below the electrodes, as well as in distant brain areas^32–34^. For dual-site right frontoparietal theta tACS during working memory, Violante and her colleagues have shown an activity increase in the stimulation targets that correlated with performance benefits^24^. Few fMRI studies focusing on resting-state imaging to investigate functional connectivity modulations through non-invasive brain stimulation over lateral parietal brain areas observed increased functional coupling between the cortical target and the hippocampus^12,18^. In the current pilot tACS-EEG study, we specifically aimed at investigating hippocampal oscillatory EEG activity. In order to quantify hippocampal theta activity, we performed subcortical source analysis^27^. Source reconstruction in EEG inherently involves assumptions and is subject to limitations. By utilizing the well-established hierarchical subspace pursuit algorithm for source reconstruction^27^ and applying rigorous procedures, we minimized the impact of noise and improved source localization accuracy. The use of individual structural MRI data allowed accurate reconstruction of each participant’s hippocampal anatomy, improving spatial precision and the reliability of the estimated neural activity.

Additionally, structural MRIs were used to record individual electrode positions, allowing a more precise calculation of the forward model, crucial for source localization. We observed a condition*memory interaction for theta (4–8 Hz) power (interaction effect: 1.25e-11, 80% CI = 0.31e-11 to 2.42e-11, semi-partial R² = 0.05), indicating that the effect of stimulation on hippocampal theta power was associated with individual memory performance during tACS. Thus, in-phase 6-Hz-tACS increased hippocampal theta power in participants with superior memory performance during the stimulation (see Fig. 1B; effect of stimulation condition (in-phase – sham) at the 75th percentile of memory performance = 4.80e-11, 80% CI = −2.86e-11 to 12.50e-11; effect at the 25th percentile = −3.65e-11, 80% CI = −11.70e-11 to 4.40e-11). More precisely, in the theta-tACS condition, higher theta power change correlated with superior memory performance. These findings suggest an involvement of hippocampal oscillatory activity in the effects of tACS which, however, may be associated to individual memory performance during tACS. The increase in theta in individuals with superior memory performance further indicates that tACS effects, including responsiveness of the subcortical system, may differ between individuals, and may depend on the capacity of higher-order cognitive functions^24,35^. We further explored the association between hippocampal theta change and memory change induced by stimulation, observing a trend towards an association between stimulation-induced changes in theta and memory (rho = 0.45, *p* = 0.07). This exploratory finding may tentatively suggest that hippocampal theta may serve as the underlying mechanism for tACS-induced memory enhancement.

## Hippocampal-cortical connectivity

Using our EEG data, we quantified the connectivity of the hippocampus with cortical brain regions where theta bursts were localized. Connectivity analyses were performed on the basis of prior studies showing the importance of the interplay of theta activity between frontal and posterior areas^36,37^, and the connection of the hippocampus within these networks^1,11^ for memory performance. The choice of the exact locations resulted from the source analysis approach that we used to identify subcortical activity (see *Methods* for further details). These included the left superior and middle frontal gyri and left temporal cortex (Fig. 1C*).* For the change in weighted phase-lag index (wPLI) between hippocampus and superior frontal cortex after stimulation, we found an interaction between stimulation condition and memory (effect: 0.007, 80% CI = 0 to 0.01, semi-partial *R*² = 0.06), suggesting that the in-phase stimulation had a beneficial effect on hippocampal-superior frontal connectivity for participants with superior memory performance [effect of stimulation condition (in-phase – sham) at 75th percentile of memory performance: 0.02, 80% CI = −0.02 to 0.06; effect of stimulation condition (in-phase – sham) at 25th percentile of memory performance: −0.03, 80% CI = −0.07 to 0.02]. Thus, similar to hippocampal theta, the connectivity between superior frontal gyri and left hippocampus was modulated depending on memory performance, with a higher effect for individuals with superior performance, suggesting an interdependency of oscillatory effects of tACS and cognitive functions. For the change in connectivity between hippocampus and middle frontal cortex, there was a stimulation effect on wPLI (mean difference between groups: −0.06, 80% CI = −0.10 to −0.02, semi-partial *R*^2^ = 0.08, see Fig. 1C, top left), suggesting decreased hippocampal-middle frontal cortex connectivity after stimulation compared to sham. This decrease reflects reduced phase synchronization between the hippocampus and a lateral prefrontal region near the stimulation target^38^. Tentatively, one can speculate that phase synchronization between the inferior frontal and angular gyri, aimed at modulating episodic memory processes mediated by the hippocampus, may reduce engagement of other higher-order networks, such as the executive control network^39^. This could result in greater segregation of the targeted memory network. There was no evidence for an effect of stimulation on wPLI between left hippocampus and left temporal cortex (effect: 0.03, 80% CI = −0.01 to 0.08, semi-partial *R*² = 0.02).

## Cortical theta power

We did not observe source-localized power changes in the left superior (mean difference between conditions: 1.14e-11, 80%-CI: −7.14e-11 9.43e-11, semi-partial R^2^ = 0.001) nor the middle frontal cortex (mean difference between conditions: 5.50e-11, 80%-CI: −7.28e-11 to 1.83e-10, semi-partial R^2^ = 0.02), but a theta power increase in the left temporal cortex in the stimulation compared to the sham condition (1.76e-10, 9.19e-11 to 2.61e-10, 0.158). This increase indicates group-level power changes in theta frequency in lateral temporal areas connected within the memory network through in-phase theta-tACS. For both middle frontal and temporal theta power, we further observed an interaction with memory performance (effect: 2.03e-11, 80%-CI = 3.48e-12 to 4.94e-11, semi-partial R² = 0.075 for middle frontal, effect: 1.55e-11, 80%-CI = 3.47e-12 to 3.78e-11, semi-partial R² = 0.044 for temporal, Fig. R1B), suggesting that the in-phase stimulation increased frontal theta power for participants with superior memory performance [for frontal theta: effect of stimulation condition (in-phase – sham) at 75th percentile of memory performance: −1.19e-10, 80%-CI = −1.97e-10 to −4.07e-11; effect of stimulation condition (in-phase – sham) at 25th percentile of memory performance: 1.85e-11, 80%-CI = −6.54e-11 to 1.02e-10; for temporal theta: effect of stimulation condition (in-phase – sham) at 75th percentile of memory performance: −2.35e-10, 80%-CI = −3.13e-10 to −1.57e-10; effect of stimulation condition (in-phase – sham) at 25th percentile of memory performance: −1.30e-10, 80%-CI = −2.14e-10 to −4.57e-11]. Thus, similar to hippocampal theta, the theta power in lateral cortical regions was modulated depending on memory performance, with a higher effect for individuals with superior performance, suggesting an interdependency of oscillatory effects of tACS and cognitive functions.

## Sources of individual variability

To explore potential sources of individual variability, we conducted two additional analyses: We examined the stimulation effects separately for different types of memory errors (e.g., false alarms vs. misses), but found no significant differences between stimulation conditions in any error category (false alarms: beta = −0.58, 80% CI = −1.55 to 0.38, semi-partial R^2^ = 0.01; misses: beta = −1.34, 80% CI = −2.69 to 0.02, semi-partial R^2^ = 0.01). We also tested for a relationship between individual differences in estimated electric field magnitude at stimulation targets (based on individual field modeling) and stimulation-related changes in memory performance. However, no significant linear associations were observed (r’s < 0.07, p’s > 0.8). While these analyses do not reveal clear subgroup effects, they help constrain interpretations and underscore the complexity of individual variability in response to stimulation.

In sum, we conducted a pilot study to explore the neural mechanisms of hippocampal oscillatory activity in response to stimulation within a controlled, within-subject design. Given our specific hypothesis regarding hippocampal involvement in tACS effects, we focused the EEG analysis on subcortical source analysis. For the first time, we present evidence that phase-synchronized tACS over lateral frontoparietal cortical areas can influence theta oscillatory activity in the hippocampus and its connectivity within the subcortico-cortical brain network. However, this effect depended on memory performance, with a stronger impact observed in individuals with superior memory performance. This finding highlights the potential role of an individual’s underlying cognitive state in determining the effects of non-invasive brain stimulation. Our pilot results provide foundational insights that can inform future confirmatory studies with larger samples; if replicated, they could pave the way for new individualized approaches in targeting hippocampal networks in health and disease, including dementia due to Alzheimer’s Disease, or temporal lobe epilepsy.

## Materials and methods

### Study design and procedure

**Fig. 2:**
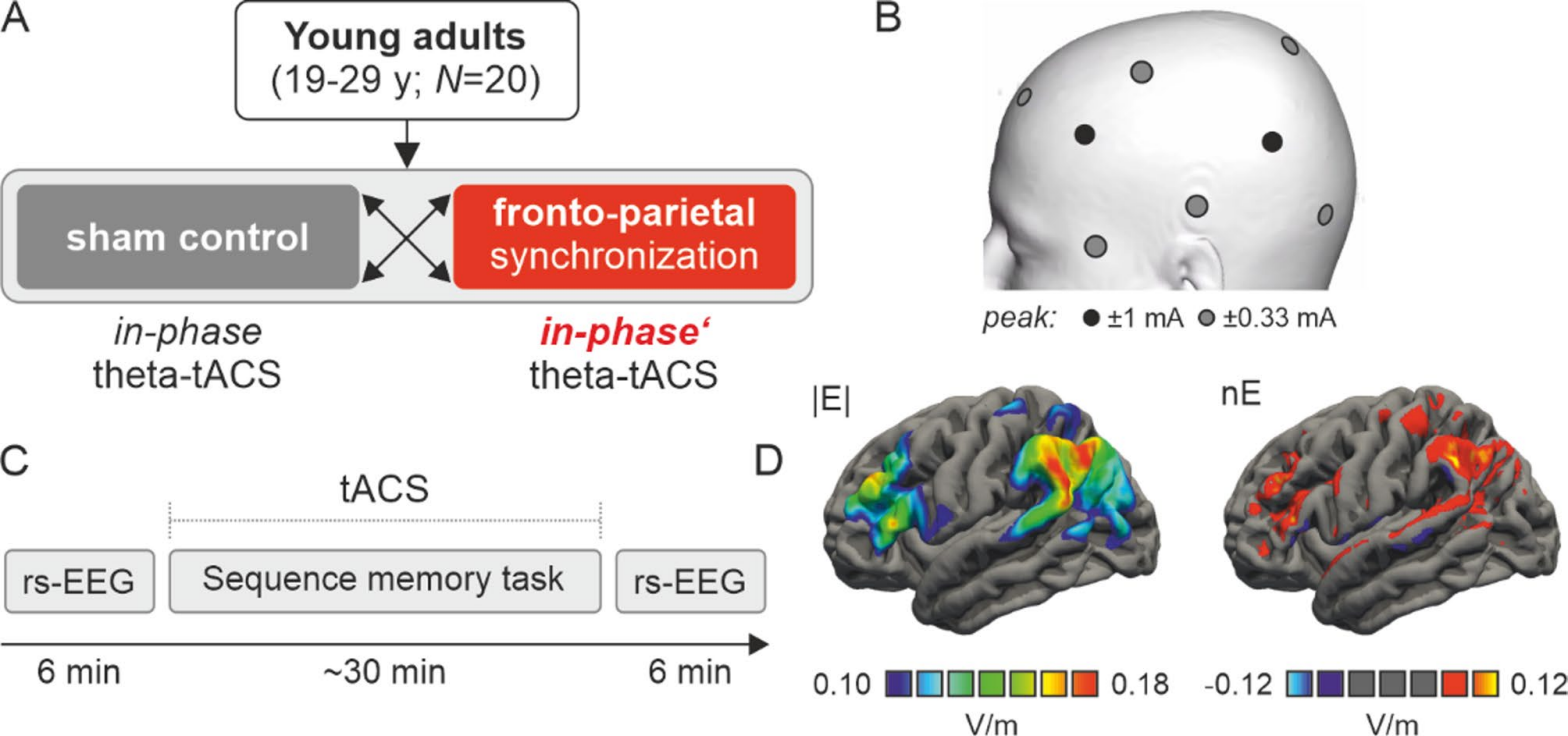
Study design, stimulation parameters, and electric field modeling. (**A**) Twenty participants (aged 19–29 years) underwent two stimulation conditions: dual-site in-phase (“synchronous”) theta-tACS (6 Hz, 30 min) and sham stimulation (30 s of in-phase tACS), applied over frontal and parietal brain regions. The order of conditions was counterbalanced across participants, with a one-week interval between sessions. (**B**) Electrodes were placed in two 3 × 1 focal set-ups with alternating current stimulation polarity (±) changes as a function of time (synchronous polarity between the central frontal and parietal electrodes). (**C**) In each session, stimulation was applied during the sequence memory task and resting-state EEG was recorded directly before and after stimulation for 6 min. (**D**) Average electric field magnitude (|E|) and the normal component of the electric field (nE) for the peak of tACS in V/m. tACS, transcranial alternating current stimulation. rs-EEG, resting-state electroencephalography.

A total of 20 participants (16 female, aged 19–29 years, mean age (SD) = 23 (3) years) were first screened via telephone and completed a structural MRI scan, along with a baseline assessment. The baseline assessment included demographic (initiated for the study) and Oldfield handedness questionnaires (sourced from^40^), as well as a practice version of the sequence memory task. Following this, transcranial alternating current stimulation (tACS) was administered either in-phase or sham (counterbalanced order) during the sequence memory task. The stimulation was delivered at 6 Hz with a peak-to-baseline intensity of 1 mA for a duration of 30 min. The study design is outlined in Fig. 2A.

All participants provided written informed consent and received reimbursement for their participation. The study was conducted in accordance with the declaration of Helsinki, and approved by the ethics committee of University Medicine Greifswald.

Resting-state EEG data were analyzed for 17 participants before and after the task (6 min each). Two participants were excluded for not meeting the performance criterion in the baseline task (below 60% correct responses), and one participant’s post-stimulation EEG recording was too noisy to detect any theta peaks.

## Structural MRI

A 3-Tesla scanner (Siemens Verio) with a 32-channel head coil was used to acquire a T1-weighted (TR = 1690 ms, TE 2.52 ms, TI = 900 ms, 176 slices, 1.0 × 1.0 × 1.0 mm^3^, flip angle 9°, selective water excitation for fat suppression) and T2-weighted (TR = 12,770 ms, TE = 86.0 ms, 96 slices, 1.0 × 266 1.0 × 1.0 mm3, flip angle 111°) structural scans of the brain.

### Task

We used an adapted version of a sequence memory task^9^ that involved 240 grayscale images of non-human objects, each presented sequentially within colored frames^41^. During the encoding phase, each image was displayed on the screen for 5000 ms, preceded by a fixation cross at the center of the screen for 500 ms and followed by a 1000 ms inter-trial interval. To promote deeper encoding, participants applied two memory strategies: (1) imagining the object in the color of the frame and indicating via button press whether the color suited the object, and (2) visualizing the current object interacting with the previous one to aid in sequence memory. After a list of five pictures, the frame color changed. During the retrieval phase, after four lists had been presented, two of the previously shown images were displayed side by side in a grey frame. Participants indicated whether the images were presented in the correct temporal order and rated their confidence using a forced-choice response with four options. For each list, images from positions one and four, and two and five, were presented in random order, resulting in eight retrieval trials per block. The correct and incorrect orders were randomized, with half of the trials in the correct order. A total of 12 blocks of images were presented, with 12 retrieval blocks, yielding 240 images in total. Two parallel versions of the task, each with different image sets, were used across the two stimulation conditions.

### tACS

Focal dual-site transcranial alternating current stimulation (tACS) was applied to target the fronto-parietal memory network using two battery-driven stimulators (DC Stimulator Plus, NeuroConn, neuroCare Group GmbH, Germany). The bipolar channels of the stimulators were split using equalizer boxes (NeuroConn, neuroCare Group GmbH, Germany) to create a 3 × 1 electrode montage. Two central Ag/AgCl stimulation electrodes (1 cm diameter) were positioned over the left hemisphere—one over the frontal cortex (centered between F3, F5, FC3, FC5) and the other over the parietal cortex (centered between P3, P5, CP3, CP5). Three surrounding electrodes for each stimulation electrode were placed on the scalp equidistantly from the stimulation electrode (frontal electrodes: in the centre of F1, F3, AFz, and AF3, in the centre of C1, C3, FC1, FC3, below the center between F7 and FT7; parietal electrodes: in the centre of CPz, CP1, Pz, P1, on PO7, in the centre of C5, T7, CP5, TP7, Fig. 2B). A current of 1 mA (peak-to-baseline) was applied for 30 min (divided in 2 × 15 min blocks with a brief rest in-between), with a 10-second ramp-up and ramp-down period. Electrode impedance was maintained below 10 kΩ. The stimulation frequency was set to 6 Hz. Two stimulation conditions were used: in-phase stimulation (0° phase, Fig. 2C) and sham stimulation (60 s of active stimulation)^20,22,24^. In order to visualize average distribution of electric fields, computational modeling was performed using SimNIBS v4.1 (simnibs.org)^42,43^. Head models were build using FreeSurfer v7^44^ and SimNIBS charm, electric field simulations were conducted using default conductivity parameters in SimNIBS. A finite element mesh was generated from T1- and T2-weighted images, including representations of the scalp, compact and spongy bone of the skull, cerebrospinal fluid, gray matter, and white matter. Electric field magnitudes and the normal component of the electric field (representing field distribution during the peak of theta-tACS) averaged across the participant group are shown in Fig. 2D.

To ensure participant blinding, a local anesthetic (EMLA^®^ cream, Aspen GmbH, Germany) was applied before the stimulation. To ensure blinding of the research staff interacting with participants, stimulation protocols for each participant and session were put in an envelope that was opened just before the stimulation by the researcher operating the EEG and stimulation device. An additional researcher not aware of the applied stimulation protocol interacted with the participant^45^.

### EEG recording

EEG was recorded at a sampling rate of 1000 Hz using a 64-channel EEG system (Brain Products GmbH, Germany). Sixty-two electrodes were placed on the scalp following the international 10–20 system. The ground electrode was positioned at FPz, and the reference electrode was placed on the tip of the nose. To monitor eye movements and blinks, two additional electrodes were placed next to the right eye and below the left eye to detect vertical and horizontal eye movements.

Impedances were kept below 5 kΩ using abrasive gel (Nuprep, Weaver and Company) for the reference and EOG electrodes, and Supervisc electrode gel was applied to all other electrodes to ensure high signal quality. Electrode positions were recorded for each participant using neuronavigation (Brainsight) and their individual MRI scans.

After electrode preparation, a 6-minute resting-state EEG was recorded while participants fixated on a cross displayed on the computer screen. Following instructions and practice trials for the sequence memory paradigm, EEG was recorded during the task (approximately 30 min) and immediately after stimulation, followed by an additional 6-minute resting-state recording.

### EEG analysis

EEG analyses were conducted using MNE Python v. 1.3 (development)^46^ on the resting-state data recorded before and immediately after the stimulation.

*Preprocessing*: Preprocessing of the EEG data consisted of resampling to 200 Hz, bandpass-filtering between 0.3 and 30 Hz (finite impulse response, one pass, zero-phase, non-causal, filter length of 11.01 s, transition bandwidth of 0.3–75 Hz, 0.0194 passband ripple, 53 dB stopband attenuation), selecting noisy channels using automated non-ocular artefact removal^47^ with our chosen threshold of 0.7, which marks as bad all channels which do not correlate with their neighbouring channels with at least *r* = 0.7. Components from independent component analysis that correlate with re-referenced vertical and horizontal EOG-channels (threshold *r* = 0.3) were rejected to remove ocular artefacts. EEG-electrode positions sampled on individual head models created from structural MRIs for each session were used as individual montages. Bursts of theta activity (4–8 Hz) were identified as follows: the resting-state data was cut into segments of 10 s to calculate a complex-valued time-frequency representation (TFR) for each channel was calculated with Morlet wavelets for frequencies from 4 to 8 Hz with 0.5 Hz increments, and wavelet cycles of 3. Power was calculated from the complex TFR, averaged across frequencies, and then transformed into *z*-scores along the time axis. Power was averaged across all channels, and then peaks were identified in the averaged power using Scipy’s *find_peaks* function. The prominence parameter was set at 1.5 after testing a series of values with several recordings for a good balance of precision and recall for theta bursts. Instantaneous phase was also calculated from the complex valued TFR and averaged across channels. This was then used to re-align the theta peaks such that they are in phase across epochs. Finally, data were epoched 400 ms before and after the identified theta peaks and only these short segments around theta bursts were used for all further analyses.

*Source analysis.* Signals from subcortical areas produce signals which are in principle strong enough to be measured on the scalp, but disentangling these from the cortically produced signals is not feasible in many circumstances, and requires careful analysis (see^27,48–50^ for recent discussions and possible solutions to the problem). We use here the approach described in^27^ which localizes the scalp signal to a sparse set of cortical sources. Dense, distributed cortical source spaces preclude the localization of subcortical sources because the range of scalp signals, they can express overlaps entirely with the range of the subcortical sources. Sparse cortical source spaces on the other hand have much less range, and may not necessarily overlap in signal space with the subcortical sources. It follows from this that if a small handful of active cortical sources can first be identified, it becomes possible also to identify a set of simultaneously active subcortical sources.

The construction of source spaces, boundary element models, and forward models were carried out in MNE Python v. 1.3 (development)^46^. Sparse/subcortical localization was done with custom code written within the MNE Python API. The structural MRIs were segmented with FreeSurfer v7.1.1^44^. First, boundary element models (BEM) and left and right hippocampal source spaces with 5-mm spacing were constructed from each participant’s MRI. Before source localization, epochs were averaged referenced, the noise covariance was calculated from the full epoch with the sample mean subtracted, and then finally averaged into (phase-locked) ERPs, one for pre stimulation and one for post stimulation. These ERPs were then input to the first, cortical stage of the subspace pursuit-based iterative greedy hierarchical algorithm, described both more generally and more thoroughly in^26^. For the first pass, the cortical surface was divided into 42 patches based on ico1 spacing. For each of the 42 patches, a leadfield matrix was calculated for all possible sources (usually several thousand) within that cortical patch. The leadfield matrix was then reduced to the number of components which explain at least 90% of the variance with Singular Value Decomposition (SVD), typically 2–3. The reduced leadfield matrices from all patches were then combined to form a dramatically reduced source space that nonetheless has nearly as much explanatory power as a full, dense cortical model. The ERP inputs were then localized into this source space using minimum norm estimation (MNE)^51^. Next, s_1_ sources were identified by calculating the norm of all source activations across time, and choosing the strongest s_1_, with the important constraint that no two sources have more than µ = 0.5 mutual coherence with each other. The reduced leadfield matrix was then restricted to the s_1_ sources, MNE localization was performed again with the s_1_ restricted leadfield matrix. The residual from this localization, along with the initial s_1_ source locations then became the initial input for the iterative, subspace pursuit process, see also^26^. This produces a sparse set of s_1_ sources, albeit with relatively poor spatial accuracy. More precision is achieved by using the sparse source locations from this step in a subsequent step.

The cortical surface is then divided into a finer set of patches based on ico2 spacing. Only the patches from the finer set which overlapped with one of the s_1_ coarser patches from the previous stage were chosen. Here our procedure differed slightly from that in^26^ as they chose not only finer patches which overlap with coarser patches, but also finer patches which border the overlapping patches^26^. An SVD reduced leadfield matrix was then calculated in the same way as above, but only for the selected, finer patches. This reduced leadfield matrix was then used to repeat the steps described above, for s_2_ sources. The resulting s_2_ sources are more accurate than the s_1_ sources from the previous step. In^26^ this process is repeated a third time with an even finer set of cortical patches^26^. Following^27^ however, we perform only two steps on the cortex, and substitute the third step with a subspace pursuit applied to the s_2_ cortical spaces from the second step combined with a subcortical source space. We first apply the same SVD reduction to the subcortical leadfield matrix, generally resulting in 2–3 sources per subcortical structure. Iterative subspace pursuit then identifies s_3_ most active sources among the sparse cortical and subcortical spaces.

*Connectivity analysis.* On the basis of prior studies showing an interplay of theta activity between frontal and posterior areas^36,37^ and the connection of the hippocampus to these networks^1,11^ we chose s_1_ = 4 and s_2_ = 4, allowing for frontal and posterior source in each hemisphere, and s_3_ = 6, allowing for a left and right source in the subcortical areas. We used the approach described in Krishnaswamy et al. (2017) which localizes the scalp signal to a sparse set of cortical sources. Dense, distributed cortical source spaces preclude the localization of subcortical sources because the range of scalp signals, they can express overlaps entirely with the range of the subcortical sources. Sparse cortical source spaces on the other hand have much less range, and may not necessarily overlap in signal space with the subcortical sources. It follows from this that if a small handful of active cortical sources can first be identified, it becomes possible also to identify a set of simultaneously active subcortical sources. Following Krishnaswamy et al. (2017), we performed two steps of the subspace pursuit-based iterative greedy hierarchical algorithm presented in Babadi et al. (2014) to identify the strongest cortical sources. After using the reduced leadfield matrix to localize sources with MNE, we selected the source from each region which had the highest amplitude, resulting finally in three cortical source time-courses for each hemisphere (superior-frontal, rostral-middle-frontal, superior/middle temporal) and one hippocampal source for each hemisphere. These eight time-courses were then input into the signal power and connectivity analyses. Here, theta band (4–8 Hz) signal power and phase coupling indexed by the weighted phase-lag index (wPLI)^52^ as a measure of connectivity were extracted from/between hippocampus, frontal and parietal cortex and used for statistical analyses.

### Statistics

The effect of in-phase stimulation was estimated using Bayesian linear mixed models (random intercept models with random intercepts for participants) for each dependent variable (% correct, “after stim” minus “before stim” difference in hippocampal theta power, “after stim” minus “before stim” difference in wPLI), adjusted for baseline performance, condition order (stim-sham, sham-stim) and visit (first or second condition). Since this was a pilot study to provide proof of concept evidence, we report effects with 80% confidence intervals (CI)^53,54^. We infer the potential presence of an effect when the CI does not encompass 0. To illustrate the direction of interaction effects, we report stimulation effects with 80% CI at the 75th and 25th percentile of memory performance. Semi-partial R² are used as effect sizes^55^.

## Electronic supplementary material

Below is the link to the electronic supplementary material.

Supplementary Material 1

## Data Availability

The datasets used and analysed during the current study are available from the corresponding author on reasonable request.
