# Supplementary material for: Memory dependent modulation of hippocampal theta power through frontoparietal phase synchronous brain stimulation
Source: Sci Rep. 2025 Jul 21;15:26504. doi: 10.1038/s41598-025-09841-y (PMC12284245; doi:10.1038/s41598-025-09841-y)
Supplement: Supplementary file 1 — Supplementary Material 1 [file 41598_2025_9841_MOESM1_ESM.docx]

**
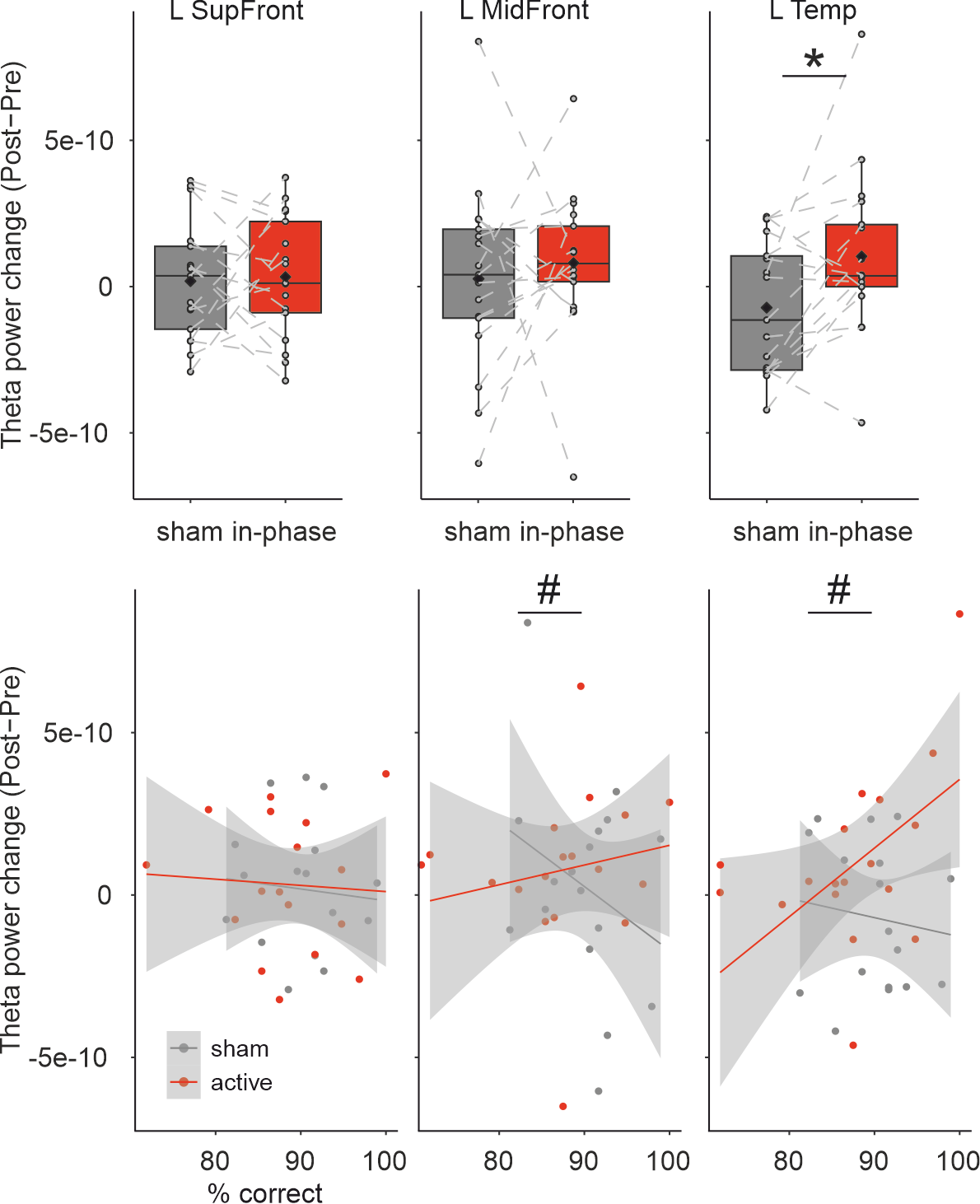
**

**Supplementary Figure 1.** **Theta power change in cortical regions after fronto-posterior tACS.** Results showed no modulation of theta power in left frontal regions, but higher increase in left temporal regions in tACS compared to sham. For middle frontal and temporal regions, there was an interaction with memory performance, indicating that the in-phase stimulation increased theta power for participants with superior memory performance. Boxplots show individual data points with the mean (diamond), median (vertical line), 25th and 75th percentiles (lower and upper hinges), and 1.5*interquartile range (lower and upper whiskers). Scatterplots show individual data points and a linear regression line with 95% confidence interval. All analyses included data of 17 participants. L SupFront, left superior frontal. L MidFront, left middle frontal. L Temp, left temporal. 80%-CI does not include 0: * main effect # interaction effect.
